# Prognostic factors for overall survival in patients with chronic myeloid leukemia treated with imatinib at the National Cancer Institute – Mexico, from 2000 to 2016

**DOI:** 10.1002/cam4.2201

**Published:** 2019-05-02

**Authors:** Jimena Ylescas‐Soria, Alfredo H. de la Torre‐Lujan, Luis A. Herrera, Daniela Miranda, Flavio Grimaldo, Silvia Rivas, Eduardo Cervera, Abelardo Meneses‐García, Fidias E. Leon-Sarmiento, Diddier Prada

**Affiliations:** ^1^ Unidad de Investigación Biomédica en Cáncer Instituto Nacional de Cancerología – Instituto de Investigaciones Biomédicas Universidad Nacional Autónoma de México (UNAM) Mexico City Mexico; ^2^ Support and Research Promotion Program (AFINES), Faculty of Mefdicine Universidad Nacional Autónoma de México (UNAM) Mexico City Mexico; ^3^ Department of Hematology National Cancer Institute Mexico City Mexico; ^4^ Department of Hematology Instituto Nacional de Cardiología "Ignacio Chávez" Mexico City Mexico; ^5^ Smell and Taste Center, Department of Otorhinolaryngology: Head and Neck Surgery, Perelman School of Medicine University of Pennsylvania Philadelphia Pennsylvania; ^6^ Mediciencias Research Group Unicolciencias/Universidad Nacional Bogota Colombia; ^7^ Departmento de Informática Biomédica, Faculty of Medicine Universidad Nacional Autónoma de México (UNAM) Mexico City Mexico

**Keywords:** chronic myeloid leukemia, hispanic, overall survival, prognostic factors

## Abstract

To determine potential predictors of long‐term survival in a large set of Hispanic (Mexican) patients with chronic myeloid leukemia (CML) treated with imatinib. We conducted an analysis with data from 411 patients with CML treated at the National Cancer Institute – Mexico, between January 2000 and December 2016. We found a median age at diagnosis of 40 years (range: 18‐84 years). The survival rate at 150 months was 82.02%, and we found that phase at diagnosis (*β*: 0.447, 95% Confidence Interval [95% CI]: 0.088, 0.806; *P* = 0.015), prognostic scales (Sokal [*P* = 0.021] and Hasford [*β*: 0.369, 95% CI: 0.049, 0.688; *P* = 0.024]) and hematological response at 3 months (*β*: 0.717, 95% CI: 0.443, 0.991; *P* < 0.001), but not molecular response (*P* = 0.834 for 6 months, *P* = 0.927 for 12 months, *P* = 0.250 for 18 months), were independently associated with overall survival. Survival analysis in subsets, according to the initial phase (chronic, accelerated and blastic phase) did not show any effect according to prognostic scales (*P* > 0.05). Mexican patients with CML have repeatedly been diagnosed at earlier ages. Prognostic factors in CML may differ according to the ethnic or geographical context. We found that phase at diagnosis, prognostic scale and hematological response at 3 months were independent predictors of survival.

## 1 INTRODUCTION

Chronic myeloid leukemia (CML) is a clonal myeloproliferative expansion of primitive hematopoietic progenitors and involves the myeloid, monocytic, erythroid, megakaryocytic and, occasionally, lymphoid lineages.^1^, ^2^ Chronic myeloid leukemia was the first human disease in which a specific abnormality of the karyotype (Philadelphia chromosome, Ph+) could be related to the pathogenetic events of leukemogenesis. The incidence of CML in the United States is 1‐2 cases per 100 000 inhabitants per year and represents 15% of leukemias in adults.^3^, ^4^ Hispanic populations typically lack accurate data; however, some previous reports suggest that CML is the type of chronic leukemia that is diagnosed most frequently.^5^ Similarly, in middle‐income Latin American countries, such as Mexico,^6^, ^7^ a majority of cases are diagnosed in the chronic phase, with an age of presentation that is usually lower than those reported in Caucasian‐population countries, impacting an age group that is in the most productive economic stage of life. Several other studies have evaluated the clinical characteristics of patients with Hispanic background diagnosed with CML.^8^, ^9^ However, we explored the roles of sociodemographic, clinical and follow‐up/treatment characteristics, including clinical responses, on long‐term survival, collected during 17 years of treatment at the National Cancer Institute (NCI) – Mexico from a large set of patients (N = 411).

## 2 MATERIALS AND METHODS

### 2.1 Study design and population evaluated

We collected relevant clinical, sociodemographic, treatment, follow‐up and response characteristics of patients with CML who were treated at the NCI – Mexico from 1 January 2000 to 31 December 2016. Socioeconomic status was obtained from the social worker's initial review and classified as follows*:* level 1 (lowest stratum) means that only 5% of the actual cost of medical care is paid, level 2 pays 10%, level 3 pays 25%, level 4 pays 53% and level 5 pays 75%. All patients received imatinib as first line of treatment. For this type of study, informed consent was not required. However, this study was approved by the Research and Ethics Committee (INCAN/CI/266/17).

### 2.2 Clinical response

The hematological response was evaluated at 3 months, and the molecular responses (MRs) were evaluated at 6, 12 and 18 months from the diagnosis date. The MR was determined from real‐time quantitative PCR analyses (qPCR), performed in peripheral blood samples, and major MR (MMR) was defined using 0.1% fusion gene containing BCR and ABL (BCR‐ABL) amplification. Complete hematological response was defined as the complete normalization of the peripheral blood count of leukocytes, the absence of immature cells, absence of symptoms and palpable splenomegaly at 3 months from diagnosis.

### 2.3 Statistical analysis

We described sociodemographic and clinical characteristics of patients, showing the number of cases per category and percentages for categorical variables. Continuous variables, such as age at diagnosis, were also categorized for descriptive purposes. We explored the association of sociodemographic and clinical characteristics with overall survival using Kaplan‐Meier plots and determined statistical significance using the log‐rank test. Finally, we explored independency for the association with overall survival using multiple Cox's regression models.

## 3 RESULTS

### 3.1 Sociodemographic and clinical characteristics

The original dataset we obtained from clinical files included 443 patients. However, 32 records (7.22%) were omitted because they lacked key clinical and sociodemographic data and information about follow‐up. Our population showed a median age at diagnosis of 40 years (range: 18‐84 years), and we found more cases in men (55.4%) than in women (44.5%). We also found that they presented with an average body mass index (BMI) of 26.5 kg/m*^2^* (SD: 4.16), with the majority of patients (41.5%) within the range of normality for BMI. The socioeconomic level that predominated was the lowest (level 1, 42.8%), followed by level 2 (33.8%; Table 1). A very good adherence to treatment (85%) was observed. Our full set of patients showed a median follow‐up time of 71.73 months. The overall survival time and the number of patients at risk at the given follow‐up times are shown in Figure S1.

**Table 1:** Sociodemographic and clinical characteristics of patients with chronic myeloid leukemia treated at the National Cancer Institute – Mexico (2000‐2016, N = 411)

Variable	N	%
Age, y		
≤40	208	50.6
41‐50	103	25.0
51‐60	63	15.3
>60	37	9.0
Gender		
Female	183	44.5
Male	228	55.4
BMI (kg/m^2^)		
Normal (18.5‐24.9)	171	41.5
Overweight (25‐29.9)	163	39.6
Obese GI	77	18.7
Socioeconomic level (SES)^a^		
1	176	42.8
2	139	33.8
3	81	19.7
4	10	2.4
5	4	0.9

Socioeconomic level: According to the Official Gazette of the Federation (DOF: 05/27/13): 1 = very low, 5 = very high.

Abbreviation: GI, Grade I; SES, socioeconomic status.

a One patient (0.4%) did not have SES recorded in clinical files.

### 3.2 Prognostic scales of patients with CML and administered doses

A majority of patients were diagnosed during the chronic phase (61.0%), followed by the accelerated phase (30.4%) and, finally, the blast phase (8.5%). In relation to the prognostic scoring scales, the highest frequency corresponded to the low‐risk category for the Sokal (45.2%), Hasford (45.7%) and Eutos (72.0%) scales. The intermediate risks category was 35.2% for the Sokal scale, 37.4% for the Hasford scale and 13.6% for the Eutos scale. The remaining percentages were for the high‐risk category Table 2. Additionally, we found that 61.5% of the patients started the treatment with 400 mg of imatinib every 24 hours, and 51.3% received a 400 mg dose at the end of the treatment Table 2.

**Table 2:** Initial phase at diagnosis, prognostic scoring scales, and imatinib dose for patients with chronic myeloid leukemia treated at the National Cancer Institute – Mexico (2000‐2016, N = 411)

Variable and categories	n	%
Initial phase		
Chronic	251	61.0
Accelerated	125	30.4
Blast	35	8.5
Prognosis risk score		
Sokal		
Low	186	45.2
Intermediate	145	35.2
High	80	19.4
Hasford		
Low	188	45.7
Intermediate	154	37.4
High	69	16.7
Eutos		
Low	296	72.0
Intermediate	56	13.6
High	59	14.3
Initial dose (mg)		
<400	62	15.0
400	253	61.5
600	84	20.4
800	12	2.9
Final dose (mg)		
<400	21	5.1
400	211	51.3
600	105	25.5
800	74	18.0

### 3.3 Molecular and hematological responses during follow‐up

We observed that the highest percentage with MMR at 12 months was 68.1%; at 18 months, this figure was 72.0%. In relation to the hematological response at 3 months, 77.8% presented a complete hematological response Table 3. From the population included at baseline, 95% of the patients were tested at 18 months for MR.

**Table 3:** Cytogenetic and molecular responses at 6, 12, and 18 months and hematological response at 3 months follow‐up for patients with chronic myeloid leukemia treated at the National Cancer Institute – Mexico (2000‐2016, N = 411)

	3 mo		6 mo		12 mo		18 mo	
	n	%	n	%	n	%	n	%
Cytogenetic response (FISH)^a^								
Complete	NA		279	67.8	206	50.1	240	58.4
Partial	NA		56	13.7	80	19.5	64	15.6
Minor	NA		51	12.4	86	20.9	87	21.2
None	NA		25	6.1	39	9.5	20	4.9
Molecular qPCR response								
Major molecular response	NA		344	83.7	280	68.1	296	72.0
No molecular response	NA		67	16.3	131	31.8	115	28.0
Hematological response^b^								
Complete	320	77.8	NA		NA		NA	
No response	61	14.8	NA		NA		NA	
Loss of response	30	7.2	NA		NA		NA	

Abbreviation: qPCR, quantitative PCR analyses; NA, data not available.

a One patient (0.4%) did not have data at 6 months. Percentages were adjusted for this missing value.

b Hematological response is evaluated only at 3 months at this institution.

### 3.4 Survival analyses

**Figure 1:**
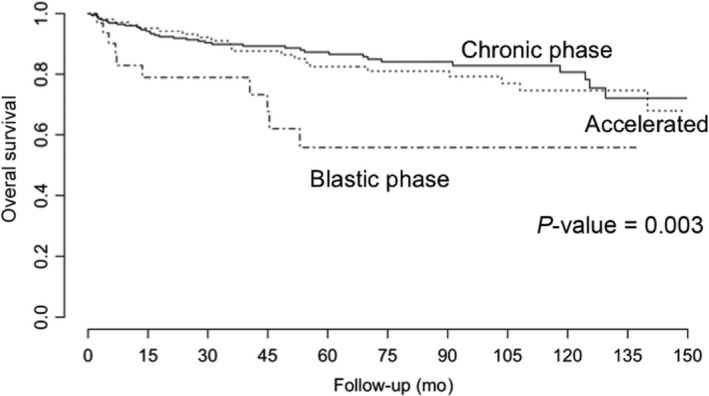
Overall survival of patients with chronic myeloid leukemia treated at the National Cancer Institute – Mexico, 2000‐2016, according to phase (chronic, accelerated and blast). *P*‐values were obtained using the log‐rank test

**Figure 2:**
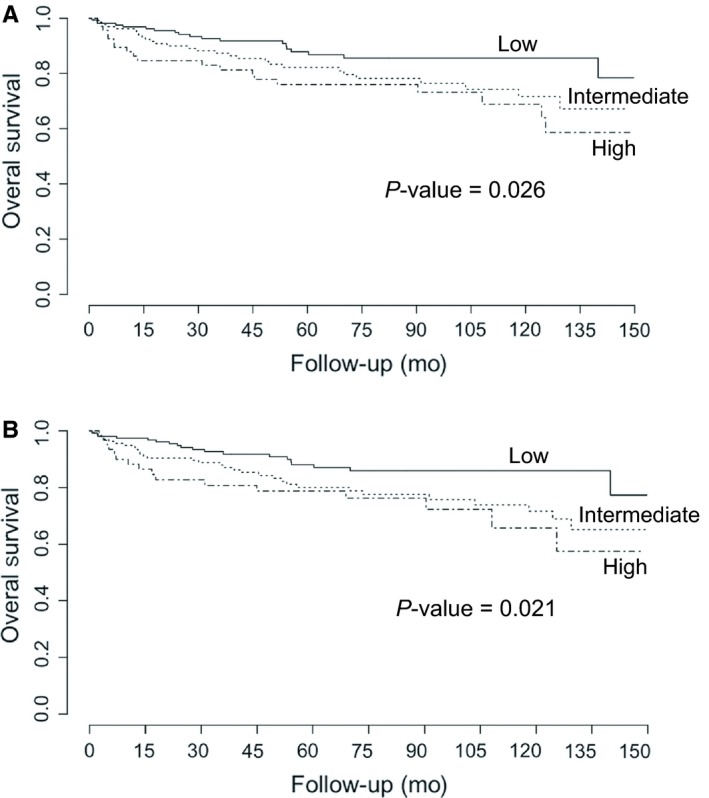
Overall survival of patients with chronic myeloid leukemia treated at the National Cancer Institute – Mexico, 2000‐2016, according to prognostic scales: A, Sokal; B, Hasford. *P*‐values were obtained using the log‐rank test

**Figure 3:**
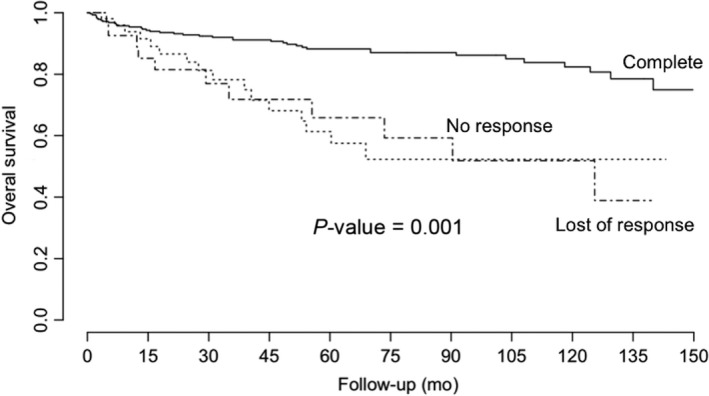
Overall survival of patients with chronic myeloid leukemia treated at the National Cancer Institute – Mexico, 2000‐2016, according to hematological response at 3 months. *P*‐values were obtained using the log‐rank test

**Figure 4:**
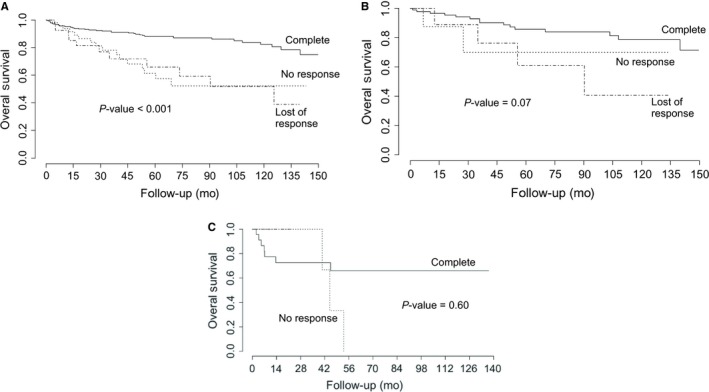
Overall survival of patients with chronic myeloid leukemia in A, the chronic phase; B, the blastic phase; and C, the accelerated phase treated at the National Cancer Institute – Mexico, 2000‐2016, according to hematologic response. *P*‐values were obtained using the log‐rank test

Survival rate in this study, at 150 months, was 82.02%. In this context, we found lower survival in patients in the blast phase, in comparison with the chronic and accelerated phases (*P*‐value = 0.003, Figure 1). Additionally, we found better survival in those patients in the low‐risk categories as per the Sokal and Hasford scales, in comparison with the intermediate and high‐risk categories (*P*‐value for Sokal: 0.026; *P*‐value for Hasford: 0.021; Figure 2). We also found that complete hematological response at 3 months was also associated with a better prognosis, in comparison with no response and loss of response categories (*P*‐value < 0.001, Figure 3). We also determined whether this effect on overall survival was dependent on phase at diagnosis and found better survival in the chronic phase when accompanied by complete hematological response (*P*‐value < 0.001). A tendency for a better survival according to hematological response was observed for patients in the blastic phase (*P*‐value = 0.07). A lack of statistical significance was observed in the group in the accelerated phase at diagnosis (*P*‐value = 0.6, Figure 4). Molecular responses (*P* = 0.834 for 6 months, *P* = 0.927 for 12 months, *P* = 0.250 for 18 months) were not associated with overall survival (Figure S2). Other factors, including age (*P*‐value = 0.77), gender (*P*‐value = 0.19), socioeconomic status (*P*‐value = 0.31), BMI (*P*‐value = 0.19), Eutos risk scale (*P*‐value = 0.49), initial doses of imatinib (*P*‐value = 0.26, Figure S3). A multiple Cox's regression model confirmed that initial phase, Hasford/Sokal scale, and hematological response at 3 months were independently associated with overall survival Table 4.

**Table 4:** Multiple Cox's regression model for the association with overall survival for patients with chronic myeloid leukemia treated at the National Cancer Institute – Mexico (2000‐2016, N = 411, 95% confidence interval [CI])

Variable	*β*	95% CI	*P*‐value
Initial phase	0.447	(0.088, 0.806)	0.015
Hasford^a^	0.369	(0.049, 0.688)	0.024
Hematological response at 3 mo	0.717	(0.443, 0.991)	<0.001

a Similar results were observed when the Sokal risk score was included in the model.

### 3.5 Survival analyses according to initial phase

As prognostic scores were initially validated for patients in the chronic phase only, we evaluated the role of both of these scores in chronic‐phase patients at diagnosis as well as in the other phases. We did not observe differences in survival according to Sokal (*P*‐values: 0.2 for chronic phase; 0.6 for accelerated phase; and 0.5 for blastic phase), Hasford (*P*‐values: 0.08 for chronic phase; 0.3 for accelerated phase and 0.8 for blastic phase) or Eutos (*P*‐values: 0.4 for chronic phase; 0.7 for accelerated phase and 0.9 for blastic phase) prognosis risk scores.

### 3.6 Survival analyses according to MR and by initial phase

We did not observe differences in survival according to MR at 6 months (*P*‐value = 0.4), 12 months (*P*‐value = 0.7) or 18 months (*P*‐value = 0.5) for patients in the chronic phase at diagnosis. For those in the accelerated phase at diagnosis, we did not observe differences according to MR at 6 months (*P*‐value = 0.2), 12 months (*P*‐value = 0.7) or 18 months (*P*‐value = 0.8). A similar trend was observed for MR in patients in the blastic phase at diagnosis (6 months, *P*‐value = 0.8; 12 months, *P*‐value = 0.9; 18 months, *P*‐value = 0.1).

## 4 DISCUSSION

In this retrospective study of patients with CML treated in a national reference center—the NCI – Mexico—we evaluated the data collected over a period of 17 years from a large number of patients treated in a public hospital from a middle‐income country, with imatinib as a first‐line treatment (pharmacological treatment donated by Novartis – Mexico). We compiled the most relevant clinical and follow‐up characteristics, including clinical and sociodemographic factors, as well their prognostic scales upon disease onset and their molecular and hematological responses. We determined that only few clinical factors, including phase at diagnosis, Sokal/Hasford risk score and hematological response at 3 months, are independent prognostic factors for long‐term survival in patients with CML.

Chronic myeloid leukemia is a serious condition; therefore, it is important to deepen our knowledge of the available prognostic factors, which may include sociodemographic characteristics, clinical characteristics and therapeutic responses, in a population with this condition but from an ethnic background different from extant studies. In a previous study in a Hispanic population conducted at a different center Hospital La Raza, Nacional Medical Center, 328 patients were investigated.^10^ In comparing percentages of response to tyrosine kinase inhibitors (TKIs, ie, imatinib) between studies, the results are dissimilar: our study had a median of age at diagnosis of 40 vs 47 years in the Hospital La Raza study; 30.4% of our patients were identified as being at the accelerated phase vs 3.05%; and 19.4% were evaluated as Sokal high risk in our study vs 35.29%. Different ages at diagnosis have also been described. For example, Faye et al reported a median age at diagnosis of 45 years in subSaharan African countries; Ben Lakhal et al reported a value of 45 years in Tunisian patients; Di Bella et al reported a median of 56 years in US patients; Latagliata et al reported a median of 58.8 years in Italian patients; Hehlmann et al reported a median of 59.3 years in US and European patients; and Nicolini et al reported a median of 61 years in patients in France. These data suggest a younger age for diagnosis according to income. The need for further research about the potential influence of socioeconomic factors on age at diagnosis in CML is a certainty. On the other hand, these studies also showed lower molecular response (MMR) in comparison with our study. We found 68.1% MMR at 12‐months; in contrast, Di Bella et al reported 38.0%, Latagliata et al reported 52.9%, and Nicolini et al reported 49.0%. These data also suggest some potential influence of external factors on MR, but further research is required.

Tyrosine kinase inhibitors have improved the prognosis of patients with CML, who reached an 82.02% survival rate at 150 months in our study, which is lower than survival rates recently reported by Hochhaus et al, who saw 83.3% after 10 years of follow‐up, but only in chronic‐phase patients.^11^ To the best of our knowledge, few studies have evaluated long‐term survival at a similar duration while including CML patients in all phases at diagnosis.

Additionally, in our study, almost 40% of patients were diagnosed as being in advanced phases of the disease, which is greater than previously published, and might be attributable to low socioeconomic status (SES), which has been associated with late medical attention.^12^ In our study, we also found a higher frequency of advanced staging at diagnosis than in other reports, which could be attributable to late medical attention and low SES in the population treated at the NCI – Mexico. However, in our analysis, SES was not related to poor survival. Further analysis about the impact of SES at the time of diagnosis, response and survival in CML is needed. When analyzing the response rate of this group of patients advance phases, we found that it was lower than the responses reached in patients in chronic phases, although this difference was not statistically significant (data not shown) and coincided with previous reports.^13^, ^14^

The prognostic scales offered by Sokal, Hasford and Eutos have traditionally been used to calculate the probability of survival in chronic‐phase CML.^15^ We found that not only prognostic scale (Sokal and Hasford, but not Eutos) but also the phase at diagnosis as well as the hematologic response at 3 months were independently associated with survival. Disease phase has been the strongest prognostic factor in CML.^16^, ^17^ In the post‐imatinib era, few studies have determined which prognostic factors may predict overall survival in CML following first‐line treatment. Our study also determined that, although prognostic scales were designed for and evaluated in chronic patients, there was no effect on survival when each phase (chronic, accelerated, blastic) was evaluated separately. To the best of our knowledge, this is the first report of prognosis factors in a Hispanic population treated exclusively with imatinib as the first line of treatment, as well as the first to evaluate effects by subpopulation (chronic, accelerated, blastic). Unfortunately, because of the low‐income status of our patients, second‐generation TKIs have not been available to be used on them.

Sokal score was initially described for patients, most of whom had been treated with busulfan.^18^ Many reports have suggested Sokal risk score, hematological response at 3 months, and cytogenetic responses at 6 and 12 months, as well as MRs at 12 and 18 months, as predictors of overall survival in CML. Although we confirmed the prognostic role of Sokal risk score (and also Hasford risk score) and hematological response at 3 months on overall survival, lack of predictive value was observed for MR, which has been found as a prognostic factor in other studies.^19^ Other studies have also determined that age, traditionally having a negative prognostic value in CML, does not have a negative impact on overall survival in imatinib‐treated patients, which was confirmed in our study.

Our study also showed lack of association between cytogenetic or MR and prognosis in CML for any of the periods evaluated (6, 12 or 18 months), including in the subanalyses according to phase. However, we found differences according to MR in those patients in the accelerated phase at diagnosis. Previous studies in CML (extensively reviewed by Hernandez‐Boluda)^20^ have suggested that cytogenetic responses at 6 and 12 months as well as MRs at 12 and 18 may be good predictors of progression‐free status and overall survival in CML patients treated with imatinib as a front‐line treatment.

Molecular response, the determination of BCR‐ABL transcripts over time, is a parameter used to determine whether CML is in remission or is persisting after TKIs treatment, and it has been proposed as an indicator to stop treatment. Molecular response is also a prognostic indicator in CML,^21^ with higher event‐free survival (EFS) rates in patients with early MMR. (Patients with BCR‐ABL transcripts >10% at 6 months and >1% at 12 months show lower EFS and higher rates of progression to accelerated phase/blast crisis, compared with other MR groups.) However, precise technical factors are required to ensure the proper determination of MR. The International Randomized Study of Interferon—STI571 (IRIS study^22^) proposed the use of the base 10 logarithm (log) for the measurement of the MR and suggested a three‐factor reduction in the log of the expression of the BCR‐ABL1 gene to define MMR. This study also highlighted the necessity of very good nucleic acid samples for reliable results.^22^ Other groups have suggested standardizing the limit of detection when MR is being determined, which was defined as the lowest detectable concentration with 95% confidence of the BCR‐ABL1 gene.^23^ Other recommendations for ensuring proper MR determination include the MIQE guidelines (Minimum Information for Publication of Quantitative Real‐Time PCR Experiments), which has shown to be useful in unifying and clarifying the results of the test.^24^ Other recommendations include obtaining more than one sample when patients show undetectable number of copies, which increases the sensitivity of the test.^23^ Based on our results and on the recent applications of cytogenetic and molecular biology laboratories in middle‐income countries, our finding might suggest the necessity of better‐quality controls, regulation and legislation to determine MR in our CML patients with reliable and reproducible results.

Our study has several limitations. First, it is a retrospective study and was limited to data obtained from medical records, thereby limiting our capacity to determine causality. However, the CML clinic at the NCI was integrated as a functional unit, with protocols of management standardized by experts in hematology and collaborators on this manuscript, thus contributing to accurate recording of the relevant medical and molecular data used in this study. Second, most patients could not receive a second‐generation TKI, even after negative response to imatinib. However, this fact homogenizes the population and allows untangling of the results of the statistical analysis. Third, most of our population had low socioeconomic status, potentially biasing our result. However, all of the patients received standard management, and imatinib as the first line of treatment. Fourth, patients in the blastic phase received chemotherapy and/or transplantation (n = 10); however, we consider the number of patients in this category to have been very low and may not have biased our final results because they corresponded to merely 8% of the cases. Additionally, this analysis did not include cytogenetics for several reasons. The NCI‐Mexico does not run conventional cytogenetics but, instead, runs fluorescent in situ hybrydization (FISH) on bone marrow samples, which misses the biological potential offered by cytogenetics (ie, analysis of the division of cells). With FISH, we could not differentiate between hematopoietic cells and those contaminating lymphocytes (usually negative for the Ph translocation) and could derive falsely negative results because of cytopenia. Additionally, we did not have access to technical information about molecular monitoring techniques run in our Institute during previous years, which may explain the lack of observed association. Finally, we also recognize that our results may be affected by survival bias, as we did not include the entire population registered in the clinical files from our institution (N = 443) during the period 2000‐2016. However, the omitted patients comprised less than 10% of the total patients, and their inclusion would limit the regression models. Lack of relevant information is common in retrospective studies and limits the generalizability of the results.

This article is the first in a series of clinical analyses that we are conducting at the NCI – Mexico, to define the greatest number of determinants (clinical and molecular factors) influencing CML in our population, including the determination of predictors of response to imatinib and the association between risk scores and hematological, cytogenetic or MRs.
