# Corrigendum

**DOI:** 10.1002/cam4.2879

**Published:** 2020-01-24

In the article by Ylescas‐Soria et al.,^1^ the Acknowledgments section was inadvertently omitted. It should be as follows:
