# Supplementary material for: Prognostic factors for overall survival in patients with chronic myeloid leukemia treated with imatinib at the National Cancer Institute – Mexico, from 2000 to 2016
Source: Cancer Med. 2019 May 2;8(6):2942–9. doi: 10.1002/cam4.2201 (PMC6558595; doi:10.1002/cam4.2201)
Supplement: Supplementary file 1 [file CAM4-8-2942-s001.docx]

**Appendices**

**Prognostic factors for overall survival in patients with chronic myeloid leukemia treated with imatinib at the National Cancer Institute - Mexico, from 2000 to 2016**

Jimena Ylescas-Soria, Alfredo H. de la Torre-Lujan, Luis A. Herrera, Daniela Miranda, Flavio Grimaldo-Gómez, Fidias León-Sarmiento, Silvia Rivas-Vera, Eduardo Cervera-Ceballos, Abelardo Meneses-García, Diddier Prada.

**Supplementary Figure legends**:

**Supplementary Figure 1.** Overall survival in the full set of patients with chronic myeloid leukemia treated at the National Cancer Institute, 2000-2016 and the number of patients at risk according to the time of follow-up (lower panel). Vertical lines correspond to censored observations.

**Supplementary Figure 2.** Overall survival in patients with chronic myeloid leukemia treated at the National Cancer Institute, 2000-2016, according to: A. Molecular response at 6 months. B. Molecular response at 12 months. C. Molecular response at 18 months. *p*-values were obtained using the log-rank test.

**Supplementary Figure 3.** Overall survival in patients with chronic myeloid leukemia treated at the National Cancer Institute, 2000-2016, according to: A. Age; B. Gender; C. Socioeconomic status (low: 1 and 2; middle: 3 and 4; high: 5, from Table 1). D. Body mass index. E. Eutos risk score. F. Initial doses of imatinib. *p*-values were obtained using the log-rank test.

Supplementary Figure 3.


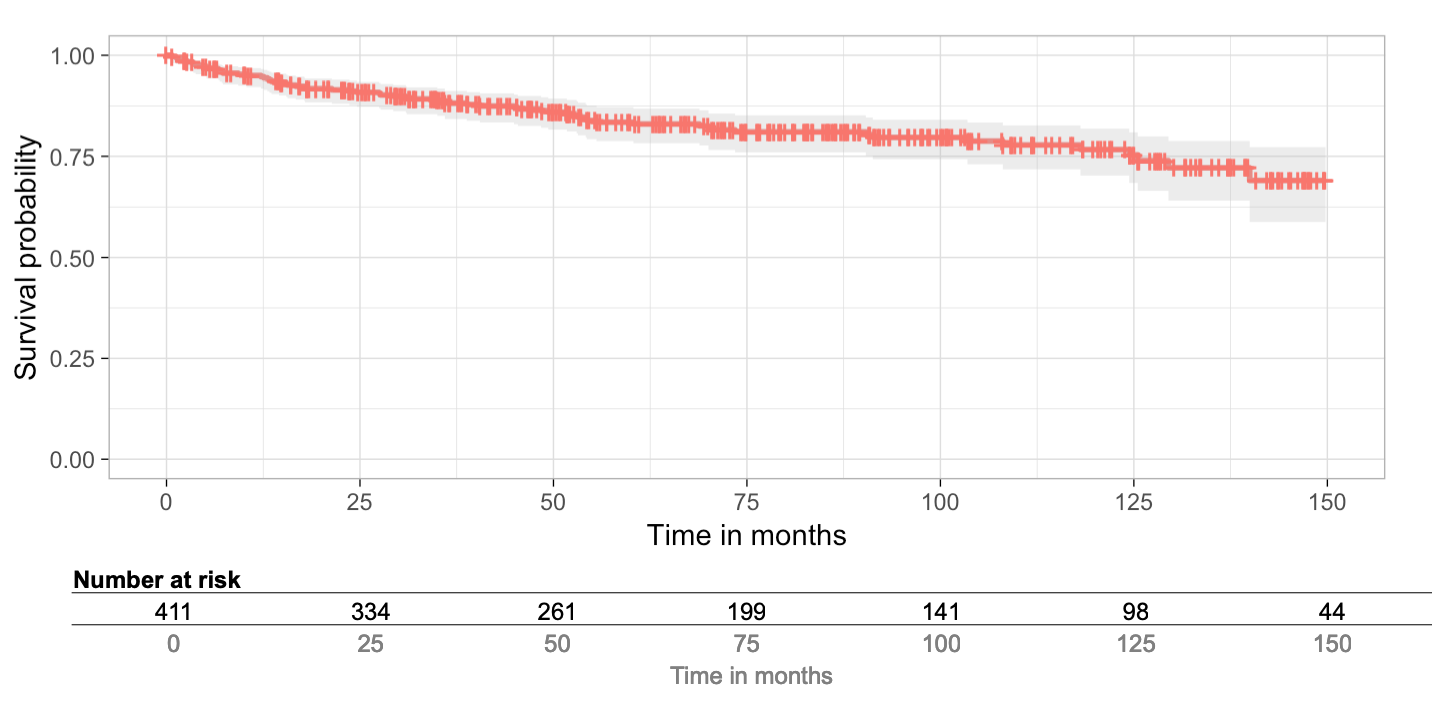


Supplementary Figure 2.

Supplementary Figure 3.
